# Malaria rapid diagnostic test transport and storage conditions in Burkina Faso, Senegal, Ethiopia and the Philippines

**DOI:** 10.1186/1475-2875-11-406

**Published:** 2012-12-06

**Authors:** Audrey Albertini, Evan Lee, Sheick Oumar Coulibaly, Markos Sleshi, Babacar Faye, Mary Lorraine Mationg, Kadi Ouedraogo, Abeba G Tsadik, Sendeaw Maksha Feleke, Ibrahima Diallo, Oumar Gaye, Jennifer Luchavez, Jessica Bennett, David Bell

**Affiliations:** 1 Foundation for Innovative New Diagnostics (FIND), Geneva, Switzerland; 2 Université de Ouagadougou, Ouagadougou, Burkina Faso; 3 Ethiopian Health and Nutrition Research Institute, Addis Ababa, Ethiopia; 4 Université Cheikh Anta Diop, Dakar, Senegal; 5 Research Institute of Tropical Medicine, Manila, Philippines; 6 National Malaria Control Programme of Senegal, Dakar, Senegal

**Keywords:** Malaria, Rapid Diagnostic Tests, RDTs, Drugs, Storage, Transport, Temperature monitoring, Stability, Temperature, Humidity

## Abstract

**Background:**

As more point of care diagnostics become available, the need to transport and store perishable medical commodities to remote locations increases. As with other diagnostics, malaria rapid diagnostic tests (RDTs) must be highly reliable at point of use, but exposure to adverse environmental conditions during distribution has the potential to degrade tests and accuracy. In remote locations, poor quality diagnostics and drugs may have significant negative health impact that is not readily detectable by routine monitoring. This study assessed temperature and humidity throughout supply chains used to transport and store health commodities, such as RDTs.

**Methods:**

Monitoring devices capable of recording temperature and humidity were deployed to Burkina Faso (8), Senegal (10), Ethiopia (13) and the Philippines (6) over a 13-month period. The devices travelled through government supply chains, usually alongside RDTs, to health facilities where RDTs are stored, distributed and used. The recording period spanned just over a year, in order to avoid any biases related to seasonal temperature variations.

**Results:**

In the four countries, storage and transport temperatures regularly exceeded 30.0°C; maximum humidity level recorded was above 94% for the four countries. In three of the four countries, temperatures recorded at central storage facilities exceeded pharmaceutical storage standards for over 20% of the time, in another case for a majority of the time; and sometimes exceeded storage temperatures at peripheral sites.

**Conclusions:**

Malaria RDTs were regularly exposed to temperatures above recommended limits for many commercially-available RDTs and other medical commodities such as drugs, but rarely exceeded the recommended storage limits for particular products in use in these countries. The results underline the need to select RDTs, and other commodities, according to expected field conditions, actively manage the environmental conditions in supply chains in tropical and sub-tropical climates. This would benefit from a re-visit of current global standards on stability of medical commodities based in tropical and sub-tropical climatic zones.

## Background

A major challenge for health systems in the tropics and sub-topics in the delivery of health care to remote and rural populations is maintaining the quality of commodities from time of entry to the country to time of use. Diagnostics and drugs can be subject to prolonged storage and transport where ambient temperature and humidity are high, while resources for environmental control are limited. The accelerated increase in the use of malaria rapid diagnostic tests (RDTs), perishable biological tests based on capture of parasite antigen by antibodies (protein) stabilized on a nitro-cellulose (organic) strip, has raised the profile of this issue within many health services in malaria-endemic regions. While emphasis on the manufacture of stable tests is essential in addressing this [1-4], understanding and managing the subsequent transport and storage of RDTs, and matching product stability to these conditions, is essential to ensure that misdiagnosis and subsequent avoidable mortality do not occur.

The WHO recommends parasitological confirmation of malaria by microscopy or alternatively by rapid diagnostic tests (RDTs) in all patients suspected of malaria before treatment initiation [5]. As most people at risk have poor access to reliable microscopy, widespread use of RDTs in remote, hard-to-supply areas is therefore essential to implement this policy [6]. Typically, RDTs and other health commodities arrive at a central warehouse, from where they may be distributed to regional or district facilities, or directly to peripheral health facilities. Recommendations on storage and transport recommend minimizing storage time at clinic level, but long peripheral storage may be necessary to maintain adequate safety stocks. A previous study in South East Asia reported storage beyond RDT manufacturer limits [7]. Such temperatures have been shown to degrade some commercially available tests [4].

Standards for registration of perishable medical commodities (drugs and *in-vitro* diagnostics) of the International Conference on Harmonization (ICH) and the Global Harmonization Task Force are well defined for cooler, temperate regions (Climatic Zones I and II) [8]. For climatic Zones III (hot/dry) and IV (hot/humid), ICH recommended a minimum stability for 1 year at 30°C and 35% humidity (Zone III) and 30°C and 65% humidity (Zone IV) [9]. While noting the ASEAN-recommended variant of 30°C and 75% humidity for Zone IV [10], the WHO still endorses the former ICH guidelines for Zones III and IV for pharmaceuticals [11], which commonly share supply lines with diagnostics. The adequacy of these standards is crucial to malaria management, as the countries concerned are often limited in their ability to control storage conditions. Furthermore remoteness and limits in capacity for managing stocks often require total product life-spans of well over the 12 months on which ICH criteria are based.

This study aimed to gather data on actual temperatures and humidity levels, in different climatic zones, to which RDTs are subjected as they move through the supply chains that typically serve malaria-endemic countries to assess the appropriateness of RDTs procured in each country programme in terms of thermal stability. With the wide roll-out of RDTs in recent years and limited capacity for quality control once RDTs are dispersed from the central warehouse, understanding the likely environment in which RDTs are transported and stored is vital for procurement decisions and patient safety.

## Methods

The study monitored temperature and humidity in storage facilities and health facilities in four malaria-endemic countries: Senegal, Burkina Faso, Ethiopia, and the Philippines. All are in ICH Climatic Zones III and IV [8], but have a range of climatic conditions. Temperature data collection was carried out during storage and transport events occurring in selected sites in each country during the study period. These were compared with the manufacturer-recommended product storage temperatures of each product at the time of the study.

### Temperature and humidity recordings

Temperatures and humidity were recorded using electronic monitors (*LogTag*® HAXO-8 Humidity & Temperature Recorder; ACR Systems, New Zealand, Range −40°C to +85°C [± 0.5°C] and 0% to 100% RH [± 3 RH]). The monitors are equipped with a button which enables users to record a “mark” in the data stream, with a corresponding date and time, to be used when an event occurs, such as when transport left, or arrived at, a storage facility. The monitors were sent to the field inside envelopes with a form on the back allowing field collaborators to record relevant events. For the purposes of this study, LogTag monitors were either stored and transported alongside RDTs, or placed in locations where RDTs are routinely stored. The monitors were set to capture temperature and humidity data every two hours in storage facilities, while those specifically sent with transport were programmed to record every 30 or 60 minutes. Stored data was downloaded from the LogTag monitors and the mean temperatures were calculated using Microsoft Excel.

### Senegal

#### Climate

Senegal, a coastal country extending into the arid Sahel, has a tropical climate with dry and rainy seasons. Average annual minimum and maximum temperatures range between 18°C and 30°C on the coast and 24°C and 40°C inland, with a maximum temperature of around 50 degrees (National Agency of Meteorology Senegal, unpublished data, 2010).

#### Supply chain and storage facilities

On delivery to the public sector, malaria RDTs are stored in the National Supply Pharmacy in Dakar, a non-air-conditioned site, prior to distribution. Temperature and humidity were monitored in the National Supply Pharmacy, two regional supply pharmacies, three district pharmacies, three health posts and one health hut (the lowest level of public-sector health care delivery). Transport data (by truck, car and bicycle) was collected from the central store to peripheral stores.

#### Study timeline

Data was collected over 13 months, from December 2009 to January 2011.

#### Malaria RDT

The test used in the National Malaria Control Programme (NMCP) during the study had a manufacturer-recommended storage temperature range from 1 to 40°C, and was detecting HRP2 (Histine-rich protein 2) antigens (single test line).

### Burkina Faso

#### Climate

Burkina Faso is a landlocked Sahel country with climatic zones ranging from very arid in the north to humid in the south, with distinct dry and rainy seasons. There is wide variation in temperatures, with an annual range between 5°C and 47°C (Institut Géographique du Burkina, unpublished data, 2011).

#### Supply chain and storage facilities

Temperatures and relative humidity were monitored in the central store (CAMEG – Centrale d’achat de Médicaments Essentiels Génériques) in Ouagadougou, one regional store where public-sector procurement is initially received, two health district stores, and three health center stores. Transport data (by van and truck) was collected from the central store to regional stores (3) and from regional stores to health district stores (3). RDTs were not kept under air-conditioning in any of these facilities.

#### Study timeline

Data was collected over a period of 12 months, from August 2009 to August 2010.

#### Malaria RDT

The RDT procured by the NMCP of Burkina Faso during the study had a recommended temperature storage range of 1°C to 40°C; this product was targeting both pLDH (*Plasmodium* lactate deshydrogenase) and HRP2 antigens (two test lines).

### Ethiopia

#### Climate

The mean annual temperatures in Ethiopia range from 10°C to 40°C, the maximum occurring in the lowlands, while the climate is milder in the central highlands, much of which are malaria-free [12].

#### Supply chain and storage facilities

Temperature and humidity were monitored in the central store (PFSA – Pharmaceutical Fund and Supply Agency) in Addis Ababa where they were temporarily stocked before distribution to three regional stores, three health center stores and six health posts in the regions. None of these facilities had air-conditioning.

#### Study timeline

Data was collected over a period of 13 months, from December 2009 to January 2011.

#### Malaria RDT

The two malaria RDTs in use during this study had storage recommendations of 4°C to 30°C and 4°C to 45°C, respectively. One product targeted both pLDH and HRP2 antigens (two test lines), while the other only HRP2 antigen (single test line).

### The Philippines

#### Climate

The Philippines is an island country with a tropical climate and a mean annual temperature of 26.6°C. During the hot season, temperatures rise above 36°C [13].

#### Supply chains and storage facilities

Temperature and humidity were monitored at the Department of Health Central Warehouse in Manila, in two regional warehouses and at health centers in three remote villages. None of these facilities was air-conditioned. Temperatures were also recorded during transport direct from the Central Warehouse to two villages.

#### Study timeline

Data was collected over a period of 15 months, from June 2009 to September 2010.

#### Malaria RDT

The malaria RDTs distributed during the study had recommended storage temperatures from 4°C to 45°C, and 4°C to 37°C, respectively. One product targeted HRP2 antigen (single test line), and the other both aldolase and HRP2 antigens (two test lines).

## Results

### Storage data

Considerable variation was recorded in temperatures throughout the commodity supply chain, with temperatures ranging from 8.3°C to 47.6°C and a maximum humidity recorded of 100% (± 3 RH). Data are summarized in Table 1.

**Table 1:** Summary table of RDT storage temperatures and humidity levels

				**% of Temp.(°C)**				
	**Min Temp. (°C)**	**Max Temp.(°C)**	**Max humidity (%RH)**	**≤ 30.0**	**>30.0**	**>37.0**	**>40.0**	**>45.0**
**Burkina Faso**	**13.6**	**42.7**	**100**					
Bobo Dioulasso -Regional Warehouse	**17.1**	**35.5**	80	61.6	38.4	0.0	0.0	0.0
Dori -Health District	**24.2**	**42.7**	73	21.1	78.9	16.3	*3.9*	0.0
Dori-Health Center	**24.2**	**41.3**	83	26.6	73.4	11.8	*1.5*	0.0
Gaoua -Health District	**13.6**	**39.4**	100	45.5	54.5	0.1	0.0	0.0
Gaoua -Health Center	**18.8**	**35.9**	100	74.4	25.6	0.0	0.0	0.0
Nouna -Health District	**17.4**	**34.9**	80	93.8	6.2	0.0	0.0	0.0
Nouna -Health Center	**20.7**	**41.8**	93	38.8	61.2	10.0	*1.2*	0.0
Ouagadougou -Central Warehouse	**26.2**	**34**	82	52.3	47.7	0.0	0.0	0.0
**Philippines**	**21.2**	**36.4**	**100**					
Cawag, Subic, Zambales City -Health Facility	**21.2**	**36.4**	100	79.9	20.1	0.0	0.0	0.0
Manila -Central Warehouse	**24.7**	**34.2**	99	76.3	23.7	0.0	0.0	0.0
Palawan -Health Facility	**23.9**	**35.8**	100	72.9	27.1	0.0	0.0	0.0
Quezon -Regional Warehouse	**23.4**	**33.6**	84	73.0	27.0	0.0	0.0	0.0
Quezon -Health Facility	**21.9**	**36.1**	98	86.4	13.5	0.0	0.0	0.0
Tuguegarao -Regional Warehouse	**22.6**	**36.4**	86	63.7	36.3	0.0	0.0	0.0
**Senegal**	**19.2**	**47.6**	**99**					
Guédiawaye -District Pharmacy	**23.9**	**32.4**	83	76.0	24.0	0.0	0.0	0.0
Dakar -National Warehouse	**20.9**	**31.3**	95	96.9	3.1	0.0	0.0	0.0
Kolda -Regional warehouse	**19.2**	**38.3**	78	66.9	33.1	1.3	0.0	0.0
Kounkané -Pharmacy/Health post	**20.6**	**37.4**	92	59.5	40.5	0.1	0.0	0.0
Lingéwal -Pharmacy/Health post	**21.5**	**40.9**	99	51.7	48.3	3.8	*0.1*	0.0
Matam -District Pharmacy	**25.4**	**40.3**	75	16.0	84.0	11.4	*0.1*	0.0
Nabadaji -Pharmacy/Health post	**23.1**	**47.6**	71	10.7	89.3	38.7	*18.2*	*1.8*
Nimzatt -Health Post	**22.6**	**34.6**	85	69.2	30.8	0.1	0.0	0.0
Ourrossogui -Regional warehouse	**22.4**	**41.8**	86	21.4	78.6	17.0	*1.4*	0.0
Velingara -District Pharmacy	**22.6**	**41.1**	95	34.8	65.2	9.1	*0.6*	0.0
**Ethiopia**	**8.3**	**43.7**	**94**					
Afar-Health center (C11)	**24.9**	**43.7**	67	19.7	*80.3*	*28.6*	*7.6*	0.0
Afar-Health post (C21)	**22.0**	41.1*	83	43.6	*56.4*	*3.7*	0.0	0.0
Afar-Health post	**25.1**	**40.9**	67	23.8	*76.2*	*14.0*	*1.0*	0.0
Afar-Regional Warehouse	**23.3**	42.2*	73	21.1	*79.0*	*1.6*	0.0	0.0
Amhara-Health post	**14.7**	**32.7**	88	99.1	*0.9*	0.0	0.0	0.0
Amhara-Health post	**16.8**	**32.5**	82	100.0	0.0	0.0	0.0	0.0
Amhara-Regional Warehouse	**17.3**	**31.3**	72	99.0	*1.0*	0.0	0.0	0.0
Amara-Health center	**19.5**	**29.6**	81	100.0	0.0	0.0	0.0	0.0
Oromia-Central Warehouse	**8.3**	**33**	94	99.1	*0.9*	0.0	0.0	0.0
Oromia-Regional Warehouse	**16.6**	**33.3**	71	100.0	0.0	0.0	0.0	0.0
Oromia-Health post (C13)	**17.6**	**30.8**	85	100.0	0.0	0.0	0.0	0.0
Oromia-Health post (C15)	**17**	**31.6**	86	100.0	0.0	0.0	0.0	0.0
Oromia-Health center	**16.2**	**29.3**	85	100.0	0.0	0.0	0.0	0.0

* Only one single temperature reading recorded above 40°C.

The temperature ranges were based on manufacturer-recommended storage temperatures for the RDTs used in this study. Percentages of temperature recorded above the recommended limit for the RDTs uses in the country are highlighted in *italics*.

### Senegal

Temperatures ranged from 19.2°C to 47.6°C. In three facilities, at the regional, district level and health center level (in the regions of Matam, Nabadaji, and Ourossogui respectively), temperatures greater than 30.0°C were recorded for more than 70.0% of the time. At the central warehouse level in Dakar, temperatures below 30.0°C were recorded 96.9% of the time. Temperatures were also reported to be higher in the Kolda regional warehouse than in the pharmacy of Kounkané, and reached a maximum of 38.3°C and 37.4°C respectively.

**Figure 1:**
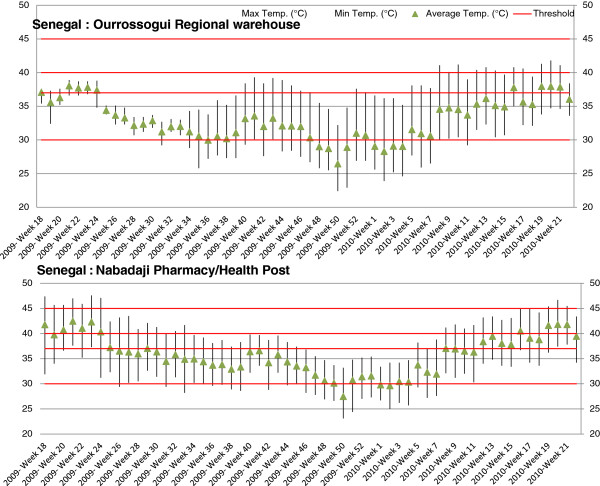
Storage temperatures across study period at 2 facilities in Senegal.

**Figure 2:**
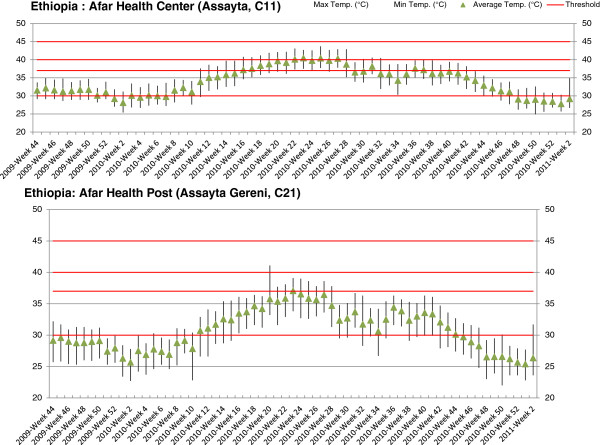
Storage temperatures across study period at 2 facilities in Ethiopia.

Temperatures were particularly high at the Ourossogui regional warehouse (maximum 41.8°C) and Nabadaji Pharmacy and Health Center (maximum 47.6°C), where temperatures above 30.0°C were recorded every week (Figure 1). Each threshold line (red lines) indicated in figures 1 and 2 represents one of the manufacturer-recommended storage temperatures for the RDTs used in this study (30, 37, 40, 45°C).

### Burkina Faso

Temperatures ranged from 13.6°C to 42.7°C. In two facilities, the health district level and health centre level in the region of Dori, temperatures higher than 37.0°C were recorded for more than 11.8% of the total storage time. At the central warehouse level in Ouagadougou, temperatures above 30.0°C were recorded 47.7% of the time. In Gaoua, temperatures were higher in the health district than in the more peripheral health centers, with maximums of 39.4°C and 35.9°C respectively.

### Ethiopia

Temperatures ranged from 8.3°C to 43.7°C. In two facilities, at health center and health post level in the region of Afar, temperatures over 40.0°C were recorded. At the central warehouse level in Addis, temperatures were over 30.0°C for 0.9 % of the time. In Oromia and Afar regions, higher temperatures were reported at the central and regional warehouses than at the heath post level. Temperatures were particularly high in Afar region where temperatures above 37.0°C were recorded over several weeks between April and October in two health centers (Figure 2).

### The Philippines

Temperatures ranged from 21.2°C to 36.4°C. At the central warehouse level in Manila, temperatures higher than 30.0°C were recorded 23.7% of the time.

### Comparison with manufacturer-recommended temperatures

In Burkina Faso, Senegal and Ethiopia storage temperatures exceeded the recommended RDT manufacturer temperature limits. In three out of eight facilities (one health district facility and two health centres) in Burkina Faso, temperatures rose above the recommended RDT manufacturer temperature limit of 40°C. In 11 out of 13 facilities in Ethiopia (one health center, six health posts, two central warehouses, two regional warehouses), temperatures exceeded the recommended RDT manufacturer temperature limit of 30°C, up to 80.3% of time at one site. In five out of ten facilities in Senegal (one regional warehouse, two district pharmacies, two pharmacies/health post), temperatures rose above the recommended RDT manufacturer temperature limit of 40°C, 18.2% of the time in one site (Table 1).

### Humidity

All RDTs are sealed in foiled packages and should not been exposed to humidity before use, although humidity is a matter of concern only when an RDT package has been damaged. Maximum humidity recorded ranges from 94 to 100% RH (Table 1).

### Transport data

Thirty transport chains were recorded for the three countries (Senegal, Burkina Faso, and the Philippines).

From the national to the regional warehouse level, two different transport chains were recorded in Senegal and each transport took 25 hours (minimum 27.3°C, maximum 38.8°C). In Burkina Faso, nine different transport chains occurred and transport took between three to nine hours (minimum 24.4°C, maximum 43°C). In the Philippines, three transport episodes were recorded from the central and regional warehouses to health centers in Palawan, Mindoro and Tuguegarao. These involved both road and air conveyances. In all three cases, the average temperature remained within acceptable limits (minimum 22.1°C, maximum 29.1°C), with the exception of one transport chain segment where the maximum temperature rose to 41.5°C. Data for all the transport chains are presented in Table 2.

**Table 2:** Transport chain conditions

**Transport chains (n)**	**Temperature range (°C)**	**Transport duration (time)**	**Comments**
**Senegal**			
National-Regional warehouse (2)	27.3-38.8	25 hours	_No cool chain system
			_Truck
Regional warehouse-Sanitary district (1)	31.6-34.4	2 hours	_ No cool chain system
			_Car
Sanitary district-Health post level (1)	31.0 (1 reading only)	1 hour	_ No cool chain system
			_Car
Health post level-health hut level (2)	31.1-42.9	4-6 hours	_ No cool chain system
			_Bicycle
**Burkina Faso**			
Central-Regional warehouse (9)	24.4-43	3-9 hours	_No cool chain system
			_Truck
Regional warehouse-health district level (7)	26.6-41.0 (no cool chain)	3-9 hours	_No cool chain system (8)
	−3.6-32 (cool chain)		_Truck
Health district-health center (4)	26.6-41.7	3-9 hours	_No cool chain system
			_Van
**The Philippines**			
Central-Regional warehouse-health center (3)	22.1-41.5	10-17 days	_No cool chain system
			_road and air conveyance
Regional warehouse-village Health center (1)	24.6-32	1 hour	_No cool chain system
			_Van

## Discussion

The malaria rapid diagnostic tests monitored in this study were exposed to conditions above manufacturer recommendations in three of the four countries studied, though for only brief periods. However, in all countries, conditions within these supply lines were above 30°C for considerable periods- beyond the WHO recommendations for stability of pharmaceuticals that are delivered through similar supply chains [11], and thus for many other commercially-available malaria RDTs. While the countries appear to have selected RDTs with stated stability, in keeping with general transport and storage conditions in this limited survey, the results raise questions over adequacy of supply line management for other medical commodities. In the four countries, drugs and RDTs are stored under the same conditions; this illustrates the importance of understanding transport and storage conditions in RDT product selection [14].

Whether the periods of temperature exposure recorded here affect RDT quality depends on the stability of the individual products. Previous studies have shown a high variability in RDT stability, both for HRP2 detecting test lines and particularly for test lines detecting pLDH [1-4]. Stability can vary at times between lots of specific products [1,3,15], but real-time stability data on a range of products lot-tested through the WHO-FIND malaria RDT evaluation programme suggests that significant failures in tests stored within manufacturer recommendations are not common [16]. The products in use by public health services in the countries involved in this study had maximum storage recommendations ranging from 40°C to 45°C, well above the former ICH recommendations, but only exceeded these temperatures for relatively short periods of time. However, these temperatures are above the recommendations for many other malaria RDTs; only 13 of the 50 RDTs tested in the Round 3 of the WHO Product Testing Programme had recommended storage temperatures of 40°C or above (FIND, unpublished data).

Other commodities (with the exception of vaccines) are generally stored together in the same facilities at central, regional, and peripheral levels. For example, certain tablet formulations of anti-malarials (e.g., artemether-lumefantrine, artesunate, amodiaquine, sulphadoxine, pyrimethamine), and HIV antiretrovirals (e.g., lopinavir, ritonavir) listed as essential drugs should not be stored above 30°C [17].

These data raise a number of issues. Firstly, diagnostics (and drugs) clearly need to be selected taking into account the expected exposure to heat and humidity. While humidity is normally addressed by the moisture-proof packaging in which the product is delivered, measuring temperature using electronic monitors is relatively easy and cheap. This knowledge should inform procurement criteria. As openly-available data on real-time stability is limited, it seems advisable for countries to require real-time heat stability data from manufacturers on which product storage recommendations should be based. It has been recommended to store RDTs at a central level for as long as possible on the assumption that peripheral storage is less controlled [18]. High temperatures recorded at central facilities in the four countries suggest that logistics planning should take actual storage data into account, and that managing conditions at central storage facilities should be taken more seriously. While storage standards for *in-vitro* diagnostic are less well defined, WHO standards for temperature stability of pharmaceuticals were exceeded at central storage over 23% of the time in two countries [11].

Clearly, published standards for pharmaceuticals and diagnostics in these climatic conditions are inadequate. Without substantial resources devoted to controlled transport and storage, temperatures of 30°C are routinely exceeded. There does appear to be a case here for investment in temperature control of central medical storage facilities. However, RDTs are a relatively high volume commodity, making controlled-temperature transport and storage at remote locations often impractical. Refrigeration is unnecessary for current products, and can also pose some risks, as shown in this study where one RDT was exposed to temperatures below −3°C in Burkina Faso. RDT storage outside the manufacturer-recommended temperature could shorten RDT product shelf life, yet this is difficult to detect. When RDTs are known to have been subjected to extreme conditions for some time, re-testing a batch withdrawn from the field against parasite panels is possible (e.g. lot testing laboratories in the Philippines and in Cambodia, [19], or comparing with microscopy, but both can be logistically difficult. Rejecting a batch on the basis of transport conditions without evidence of poor performance is costly. The development of positive control wells, based on lyophilized parasite antigens, holds promise to address clinic-level quality control dilemmas for malaria RDTs [20].

Lower cost solutions are available for remote-area storage, such as underground storage or the use of evaporative cooler boxes [21]. Storage using evaporative cooling has been demonstrated for malaria RDTs in Afghanistan [22] and Cambodia [23], reducing temperatures from as much as 37°C to 23°C without use of electricity, well within the common range for storage of non-vaccine medical commodities. Moreover, simple measures during transport, such as loading and discharging vehicles in the shade or at night, reduce the probability of deterioration [21,24]. In the end, the performance of the product at end-user level is the most important measure of impact of previous environmental damage; this is difficult to sustain for both diagnostics and drugs. Whereas this is currently being addressed for malaria, monitoring the availability of the active ingredient of a drug in a remote location is likely to remain a major challenge.

Due to the complexities of coordinating logistics throughout transport chains, only segments of the transport chain were recorded in this study. A larger study would likely reveal a wider range of conditions and problems. Since only a small sample of transport and storage was collected in each country, the results cannot be considered fully representative of the conditions under which medical commodities are transported and stored in any of these countries. However, they illustrate the lack of control in supply chains and potential for environmental damage of products. Ideally, the condition of RDTs should also have been tested; the countries concerned procure RDTs with relatively high recommended maximum storage temperatures so it is unclear whether the relatively short periods when they are exposed to higher temperatures would have been significant. However, the study results raise concern with respect to RDT stability between production lots (pre-release real-time testing of production lots is obviously not possible [1,3], as well as the effect of their co-storage with other perishable medical commodities, as discussed above.

## Conclusion

Transport and storage temperatures varied widely throughout the supply chains monitored here. In particular high temperatures were recorded at central storage facilities in some countries. While the recommended storage conditions of the RDTs used by these health services were rarely exceeded, conditions were inappropriate for many of the RDTs on the market and frequently exceeded common pharmaceutical storage standards. A number of simple measures can be instituted to lower ambient temperatures, and tests for commodity quality control are clearly needed. The results illustrate the importance of health services understanding their supply chain conditions and matching product procurement to these. They also suggest that a revisit of standards is indicated for medical commodities in general in the tropics and sub-tropics, as existing standards for Climatic Zones III and IV appear inadequate on the basis of this study.

## Competing interests

The authors declare they have no competing interests.

## Authors’ contributions

AA, DB and EL designed the study protocol and instructions. MM, KO, ID, OG, AG and SM carried out the monitoring and data collection process. AA and EL carried out the analysis. AA, SOC, BF, MS, JB, DB and EL drafted the manuscript. All authors have read and approved the manuscript.
